# Characteristics of High-density Lipoprotein Subclasses Distribution for Subjects with Desirable Total Cholesterol Levels

**DOI:** 10.1186/1476-511X-10-64

**Published:** 2011-04-22

**Authors:** Li Tian, Shiyin Long, Mingde Fu, Yinghui Liu, Yanhua Xu, Lianqun Jia

**Affiliations:** 1 Laboratory of Endocrinology and Metabolism, West China Hospital, Sichuan University, Chengdu 610041, Sichuan, People's Republic of China; 2 Department of Biochemistry and Molecular Biology, University of South China, Hengyang, Hunan, People's Republic of China; 3 Chengdu Hoist Biotechnology Co., LTD, Sichuan, PR China

## Abstract

**Background:**

To investigate alteration of high density lipoproteins (HDL) subclasses distribution in different total cholesterol (TC) levels, mainly the characteristics of HDL subclasses distribution in desirable TC levels and analyze the related mechanisms.

**Methods:**

ApoA-I contents of plasma HDL subclasses were determined by 2-dimensional gel electrophoresis coupled with immunodetection. 486 Chinese Adults subjects were assigned to different TC groups according to the third Report of NCEP (ATP- III) guidelines.

**Results:**

The increase in contents of small preβ_1_-HDL, HDL_3c_, HDL_3b_, and HDL_3a _particles clustered and reduce in HDL_2b _with increased of TC. The distribution of HDL subclasses have shown abnormality characterized by the lower HDL_2b _(324.2 mg/L) contents and the higher preβ_1_-HDL (90.4 mg/L) contents for desirable TC Chinese subjects. Among 176 desirable TC subjects, 58.6% subjects with triglyceride (TG) < 2.26 mmol/L, 61.2% subjects with HDL-C ≥1.03 mmol/L and 88.6% subjects with low density lipoprotein cholesterol(LDL-C) < 3.34 mmol/L, and the profile of HDL subclasses distribution for above these subjects was reasonable.

**Conclusions:**

The particles size of HDL subclasses shifted towards smaller with increased TC levels. The TC was liner with HDL_2b _contents and those can be reduced 17 mg/L for 0.5 mmol/L increment in TC levels. The HDL subclasses distribution phenotype was not expectation for Chinese Population with desirable TC levels. Thus, from the HDL subclasses distribution point, when assessing the coronary heart disease(CHD) risk not only rely on the TC levels, but also the concentrations of TG, HDL-C and LDL-C must considered in case the potential risk for desirable TC subjects with other plasma lipids metabolism disorders.

## Introduction

Cholesterol is a fat-like substance used to help build cell membranes, make some hormones, and form bile secretions that aid in digestion. cholesterol's most important job is to help carry fat through your blood vessels. Cholesterol travels in the blood in distinct particles containing both lipid and proteins (lipoproteins). Classical concepts of the regulation of plasma cholesterol levels involve roles for the "forward" delivery of low density lipoprotein cholesterol (LDL-C) from the liver to the peripheral tissues, mediated by the LDL receptor, and a "reverse" delivery of cholesterol in the form of high density lipoproteins (HDL) from the peripheral tissues to the liver [1]. The amount of HDL and LDL in the blood is added together, this number for all practical purposes, indicates the amount of total cholesterol (TC).

LDL-C is the primary transport carrier of cholesterol in the circulation. A broad base of evidence indicates that elevations in LDL cholesterol are a direct cause of atherosclerosis. Long-term elevations of LDL cholesterol lead to a progressive accumulation of coronary atherosclerosis [2]. Thus, LDL-C is proposed to be more highly associated with coronary heart disease (CHD) [3].

HDL-C normally makes up 20-30% of the TC and it prevents the uptake of LDL-C at receptor sites in the body and participates in the metabolism of other lipoproteins. Strong epidemiological evidence links low levels of serum HDL cholesterol to increased CHD morbidity and mortality. High HDL-C levels conversely convey reduced risk [4-6]. Epidemiological data taken as a whole signify that a 1 percent decrease in HDL cholesterol is associated with a 2-3 percent increase in CHD risk.

HDL is highly heterogeneous, with subclasses that can be identified on the basis of density, size, charge, and protein composition [7] and using two-dimensional gel electrophoresis coupled with immunoblotting, HDL can be divided into large, cholesterol-rich (HDL_2a _and HDL_2b_), small, lipid-poor (HDL_3c_, HDL_3b_, HDL_3a_, and preβ_1_-HDL) and preβ_2_-HDL. We have previously investigated the impact of plasma triglyceride (TG), TC, LDL-C and HDL-C levels on HDL subclass distribution, and found that the particle size of HDL shifted towards smaller size as the rise of plasma TG, TC, LDL-C levels, or the fall of HDL-C levels [8-11].

Thus, in this work, we mainly assessed that the effect of TC levels on HDL subclasses distribution, especially the characteristics of HDL subclasses distribution for subjects with desirable TC levels [12].

## Materials and methods

### Subjects

Four hundred and eighty-six subjects, aged 33 to 78 years (56.1 ± 8.4), were recruited to participate in a study examining plasma lipid and apolipoprotein concentrations. were recruited to participate in a study examining plasma lipid and apo concentrations at West China Medical Center, Sichuan University. These subjects were from the Sichuan University and Sichuan Normal University, in Chengdu, Sichuan province, PR China, in which female were 198 and male were 288. Exclusion criteria were the following: (1) the presence of nephrosis, diabetes mellitus, hypothyroidism, or hepatic impairment; (2) the presence of a major cardiovascular event (myocardial infarction, severe or unstable angina pectoris, and surgery) or stroke; (3) taking lipid-altering medications; or (4) a history of alcohol abuse and smoking cigarettes. Women who had undergone a hysterectomy with or without an oophorectomy were excluded from the study, and for postmenopausal female subjects, none were receiving hormone replacement therapy. Informed consent was obtained from each subject upon entry into the study population. This study protocol was approved by the ethics committee.

To study the relationship between the TC levels and HDL subclass distribution, we divided these subjects into three subgroups using the Adult Treatment Panel(ATP-III) guidelines for TC defined, that is, desirable TC (<5.17 mmol/L), borderline-high TC (5.17-6.18 mmol/L), high TC (≥ 5.17 mmol/L) [12]. Moreover, individuals were classified according to approximately equal ninths of baseline TC for the entire study population, and observed the levels of TC change and degree of major HDL subclasses alteration.

### Specimens

Whole blood specimens were drawn after a 12 hours overnight fast into EDTA-containing tubes. Plasma was separated within 1-2 hour. Plasma was stored at 4°C and used for lipid and apo analyses within 24 hours. An aliquot of plasma was stored at -70°C for the determination of HDL subclasses.

### Plasma lipid and apolipoprotein analyses

Plasma TG, TC and HDL-C concentrations were measured by the standard technique. TC and TG were determined with enzymatic kits (Beijing Zhongsheng Biotechnological Corporation, Beijing, and People's Republic of China). The HDL-C was determined after precipitation of the apoB-containing lipoproteins by phosphotungstate/magnesium chloride [13]. LDL-C was calculated using the Friedwald formula (TG < 4.52 mmol/L) [14]. When plasma TG was at least 4.52 mmol/L, LDL-C was determined following the precipitation method with polyvinylsulfate (enzymatic kits). Plasma apoA-I, B-100, C-II, and C-III were determined by radial immunodiffusion methods [15] using kits developed at the Apolipoprotein Research Laboratory, West China Medical Center, Sichuan University. The intraassay coefficient of variation for apo concentrations was between 2.1% and 4.8%; the interassay coefficient of variation was 3.5% to 7.9% [16].

### High density lipoprotein cholesterol subclass analysis

ApoA-I-containing HDL subclasses were measured by nondenaturing two-dimensional gel electrophoresis associated with the immunodetection method, as described previously [17]. Briefly, 10 μl of plasma was first separated by charge on 0.7% agarose gel into preβ and α mobility particles. In the second dimension, the two fractions of HDL were further separated according to size by 2-30% nondenaturing polyacrylamide gradient gel electrophoresis. To determine HDL subclasses, Western blotting was conducted after 2-D gel electrophoretic, plasma proteins and molecular markers were electroretically transferred to PVDF membranes, stained with 0.1% ponceau S, and the position of molecular standard protein bands labeled by pencil, and destained by diffusion, then using 5% bovine serum albumin(BSA) recovered the membrane, following interaction with horseradish peroxidase (HRP)-labeled goat antihuman apo A-I immunoglobulin G. The HDL particle sizes were calibrated using a standard curve that included bovine serum albumin, ferritin and thyroglobulin (Pharmacia, Uppsala, Sweden). The calculation of the relative percentage of each HDL subclass was based on the density of the electrophoresis spots. Then, the apoA-I contents (in milligrams per liter) of the HDL subclasses were calculated by multiplying the percentage of each subclass by the plasma total apoA-I concentrations. The interassay coefficients of variation of the relative concentration of preβ_1_-HDL, preβ_2_-HDL, HDL_3c_, HDL_3b_, HDL_3a_, HDL_2a_, and HDL_2b _in the plasma sample were 9.4%, 9.8%, 4.9%, 6.2%, 7.3%, 11.1% and 7.9%, respectively (n = 5).

### Statistical analysis

Kolmogorov-Smirnov statistics was used to test normality of distributions of plasma lipids and HDL subclasses. For analysis, non-Gaussian-distributed data were transformed using the natural logarithm to approach a Gaussian distribution. All statistical analyses were performed using the statistical package SPSS Version 13.0 (SPSS, Chicago, IL). Data in the tables are expressed as mean ± S.D. The Duncan post-hoc test was used in situations in which a significant group (one-way analysis of variance) effect was observed. Two-way ANOVA was performed to evaluate the interactive effects of TG and TC on HDL subclasses distribution. Pearson correlation coefficients were calculated to quantitatively analyze the associations among variables adjusted for TG concentration. In all comparisons, *P *less than 0.05 (2-sided) represented a statistically significant difference.

## Results

### Relations of the preβ_1_-HDL and HDL_2b _contents and change in the levels of TC

**Figure 1:**
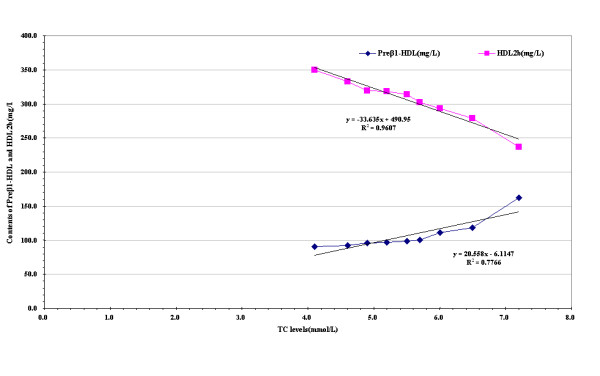
**Relations of the preβ_1_-HDL and HDL_2b _contents to the levels of TC**.

To investigate the degree of major HDL subclasses change with the levels of TC, varied, we divided the levels of TC into 9 strata and using each stratum TC median value as x axis, with every stratum TC corresponding the median preβ_1_-HDL and HDL_2b _contents as y axis to plot (Figure 1). The figure shows associations of TC levels with decreased HDL_2b _contents.

### The concentrations of plasma lipids, lipoproteins, and apolipoproteins along with their ratios among subjects categorized by TC concentration

Table 1 reports baseline demographic and plasma levels of lipids, lipoproteins, and apolipoproteins as well as their ratios for subjects in each TC subgroup. Subjects in desirable and borderline-high TC subgroups were somewhat younger than those included in high TC subgroup. Concentrations of TG, TC, LDL-C, HDL-C, and apoB-100, C-II, C-III along with the ratios of TC/HDL-C, LDL-C/HDL-C and apoB-100/A-I were substantially lower in desirable and borderline-high TC subgroups than those in high TC subgroup.

**Table 1:** The concentrations of plasma lipids, lipoproteins, and apolipoproteins their ratios among subjects categorized by TC levels

	Desirable TC (n = 176)	Borderline high-TC (n = 194)	High TC (n = 116)
**Age(yr)**	54.9 ± 8.3	57.2 ± 9.3^a‡^	58.6 ± 9.2^a§^
**BMI(kg/m**^**2**^**)**	23.3 ± 2.4	23.8 ± 2.3	23.2 ± 2.9^a§ b§^
**TG(mmol/L)**	1.9 ± 0.2	2.2 ± 0.4	2.4 ± 0.5^a‡^
**TC(mmol/L)**	4.6 ± 0.4	5.6 ± 0.3^a‡^	6.8 ± 0.4^a§ b‡^
**LDL-C(mmol/L)**	2.5 ± 0.4	3.3 ± 0.6^a‡^	4.4 ± 0.7^a§b^*
**HDL-C(mmol/L)**	1.1 ± 0.2	1.2 ± 0.3	1.3 ± 0.4^a^*
**ApoA-I(mg/L)**	1210.7 ± 168.6	1282.2 ± 179.1	1280.8 ± 171.6
**ApoB-100(mg/L)**	769.6 ± 96.4	925.3 ± 108.3^a‡^	1081.4 ± 128.2^a§ b‡^
**ApoC-II(mg/L)**	55.1 ± 10.3	70.1 ± 12.7^a^*	79.2 ± 11.8^a^*
**ApoC-III(mg/L)**	129.1 ± 20.6	157.5 ± 22.6^a^*	180.2 ± 32.6^a§b^*
**TG/HDL-C**	2.0 ± 0.3	2.2 ± 0.2	2.2 ± 0.3
**TC/HDL-C**	4.2 ± 1.0	5.0 ± 1.0^a‡^	5.5 ± 1.1^a§b^*
**LDL-C/HDL-C**	2.3 ± 0.4	2.9 ± 0.7^a^*	3.5 ± 0.9^a§b‡^
**ApoB-100/A-I**	0.7 ± 0.2	0.7 ± 0.2	0.9 ± 0.3^a* b^*

Values are expressed as Mean ± S.D

^a^Compared with Desirable TC

^b^Compared with Borderline high-TC

*P < 0.05, ^‡^*P *< 0.01, ^§^*P *< 0.001

TC, total cholesterol; BMI, body mass index; TG, triglyceride; LDL-C, low density lipoprotein cholesterol; HDL-C, high density lipoprotein cholesterol; ApoA-I, B-100, C-II, C-III, apolipoproteinA-I, B-100, C-II, C-III.

### The apolipoprotein A-I contents of HDL subclasses according to the TC concentration

Table 2 displays the characteristics of HDL subclasses contents for subjects in per TC subgroup. We observed that increases in the contents of small preβ_1_-HDL, HDL_3c_, HDL_3b_, and HDL_3a _particles clustered with the increases in TC concentration. When compared with the subject in high TC subgroup, the subjects in both borderline-high and desirable TC subgroups had higher contents of large HDL_2a _and HDL_2b _particles. However, large sized HDL_2 _particles kept the lower levels for subjects in desirable TC subgroup.

**Table 2:** The apoA-I contents of HDL subclasses among the subjects according to the TC levels

	Desirable TC (n = 176)	Borderline high-TC (n = 194)	High TC (n = 116)
**Preβ**_**1**_**-HDL(mg/L)**	90.4 ± 15.3	102.2 ± 20.8	135.8 ± 24.9^a§b^*
**Preβ**_**2**_**-HDL(mg/L)**	53.5 ± 8.4	63.4 ± 9.1	70.0 ± 11.6^a^*
**HDL**_**3c**_**(mg/L)**	69.1 ± 7.6	73.9 ± 8.6	82.7 ± 10.5
**HDL**_**3b**_**(mg/L)**	135.9 ± 21.5	145.6 ± 22.3	172.7 ± 28.6^a‡b^*
**HDL**_**3a**_**(mg/L)**	281.9 ± 29.2	298.2 ± 24.2^a^*	319.3 ± 30.1^a§b^*
**HDL**_**2a**_**(mg/L)**	257.2 ± 20.1	259.9 ± 18.3	251.6 ± 21.5
**HDL**_**2b**_**(mg/L)**	324.2 ± 30.3	337.5 ± 35.3	281.7 ± 29.9^a§b§^

Values are expressed as Mean ± S.D

^a^Compared with Desirable TC group

^b^Compared with Borderline high-TC group

*P < 0.05, ^‡^*P *< 0.01, ^§^*P *< 0.001

TC, total cholesterol; HDL_3c_, _3b_, _3a_, _2a_, _2b_, high density lipoproteins_3c_, _3b_, _3a_, _2a_, _2b_.

### Frequency distribution of the subjects according to the concentrations of TG and HDL-C for subjects with desirable TC levels

**Figure 2:**
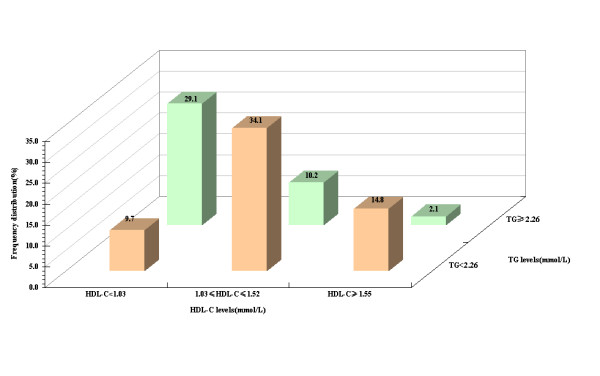
**Frequency distribution of subjects according to HDL-C together with TG levels**.

To further understand the profile of HDL subclasses distribution in different levels of TG and HDL-C for subjects with desirable TC, we using the ATP-III set the 1.03, 1.52 mmol/L for HDL-C and 2.26 mmol/L for TG as cut-points to divided these subjects (Figure 2).

Figure 2 presented that there were 51 subjects (29.1%) with TG ≥ 2.26 mmol/L-HDL-C < 1.03 mmol/L, and only 14.8% (26) subjects with TG < 2.26 mmol/L-HDL-C ≥ 1.55 mmol/L in desirable TC levels. Moreover, in the desirable TC levels, the subjects with TG ≥ 2.26 mmol/L occupied approximately 41.4%, and 38.8% subjects with HDL-C < 1.03 mmol/L.

### The apolipoprotein A-I contents of HDL subclasses according to the TG or HDL-C concentrations in desirable TC subjects

As shown in Table 3, in desirable TC subjects, the contents of preβ_1_-HDL, HDL_3b_, and HDL_3a _in TG < 2.26 mmol/L subgroup were significantly lower than TG ≥ 2.26 mmol/L subgroup (*P *< 0.001). In contrast, those of HDL_2a _and HDL_2b _in TG < 2.26 mmol/L subgroup were significantly higher than TG ≥ 2.26 mmol/L subgroup (*P *< 0.001). Likewise, compared to the subjects with HDL-C < 1.03 mmol/L, the characteristics of HDL subclasses distribution in subjects with HDL-C ≥ 1.03 mmol/L was accord with that in subjects with TG < 2.26 mmol/L.

**Table 3:** The apoA-I contents of HDL subclasses among the desirable TC subjects in accordance with TG along with HDL-C levels

	Desirable TC		Desirable TC	
	TG < 2.26(n = 103)	TG ≥ 2.26(n = 73)	HDL-C < 1.03(n = 69)	HDL-C ≥ 1.03(n = 107)
**Preβ**_**1**_**-HDL(mg/L)**	79.3 ± 10.7	118.1 ± 21.4^§^	115.4 ± 11.5	83.1 ± 8.1^‡^
**Preβ**_**2**_**-HDL(mg/L)**	50.6 ± 8.1	56.1 ± 9.2	53.4 ± 4.2	54.5 ± 4.7
**HDL**_**3c**_**(mg/L)**	63.6 ± 8.6	72.2 ± 10.9	72.2 ± 8.4	64.0 ± 5.8
**HDL**_**3b**_**(mg/L)**	105.2 ± 28.9	146.9 ± 30.2^§^	146.5 ± 19.6	128.4 ± 17.4
**HDL**_**3a**_**(mg/L)**	258.2 ± 30.4	315.3 ± 40.9^§^	297.6 ± 28.9	274.1 ± 20.5*
**HDL**_**2a**_**(mg/L)**	268.6 ± 35.3	226.7 ± 29.7^§^	222.8 ± 20.7	272.6 ± 21.5^§^
**HDL**_**2b**_**(mg/L)**	381.9 ± 50.5	242.8 ± 29.9^§^	247.6 ± 25.1	371.3 ± 40.6^§^

Values are expressed as Mean ± S.D

Compared with the corresponding low levels group

*P < 0.05, ^‡^*P *< 0.01, ^§^*P *< 0.001

TC, total cholesterol; TG, triglyceride; HDL_3c_, _3b_, _3a_, _2a_, _2b_, high density lipoproteins_3c_, _3b_, _3a_, _2a_, _2b_.

### The apolipoprotein A-I contents of HDL subclasses according to the LDL-C concentrations in desirable TC subjects

Table 4 showed that in comparison with the subjects with LDL-C < 3.34 mmol/L, the subjects with LDL-C ≥ 3.34 mmol/L had higher contents of HDL_3b_, HDL_3a _and lower contents of HDL_2a _and HDL_2b _in desirable TC levels.

**Table 4:** The apoA-I contents of HDL subclasses among the desirable TC subjects in accordance with LDL-C levels

	Desirable TC	
	LDL-C < 3.34 (n = 156)	LDL-C ≥ 3.34 (n = 20)
**Preβ**_**1**_**-HDL(mg/L)**	84.3 ± 8.0	110.8 ± 8.2*
**Preβ**_**2**_**-HDL(mg/L)**	50.8 ± 3.2	53.6 ± 3.7
**HDL**_**3c**_**(mg/L)**	59.1 ± 5.4	69.6 ± 6.1
**HDL**_**3b**_**(mg/L)**	135.3 ± 18.9	153.2 ± 20.1*
**HDL**_**3a**_**(mg/L)**	263.5 ± 26.9	282.8 ± 29.2*
**HDL**_**2a**_**(mg/L)**	288.1 ± 27.5	249.5 ± 20.4^§^
**HDL**_**2b**_**(mg/L)**	370.6 ± 39.9	252.0 ± 30.8^§^

Values are expressed as Mean ± S.D

Compared with the corresponding low levels group

*P < 0.05, ^§^*P *< 0.001

TC, total cholesterol; LDL-C, low density lipoprotein cholesterol; HDL_3c_, _3b_, _3a_, _2a_, _2b_, high density lipoproteins_3c_, _3b_, _3a_, _2a_, _2b_.

### Relationships among plasma lipids, lipoproteins, apolipoproteins and HDL subclasses contents (Controlling for TG)

After adjustment for TG, the TC concentration was positively related to preβ_1_-HDL, preβ_2_-HDL, HDL_3 _(HDL_3c_, HDL_3b_, and HDL_3a_) and negatively related to HDL_2 _(HDL_2a _and HDL_2b_). The relationship for LDL-C and apoB-100 showed a significant positive with preβ_1_-HDL, HDL_3c_, HDL_3b _but inverse with HDL_2b_. The apoA-I levels were strong association with all HDL subclasses. Meanwhile, a significant correlation between the preβ_1_-HDL, HDL_3c_, HDL_3b_, HDL_2 _and HDL-C was observed (Table 5). The 2-way ANOVA results indicated that there were significant interaction of TC and TG effect on preβ_2_-HDL, HDL_3c_, HDL_3b_, HDL_3a_, and HDL_2a_(Table 6).

**Table 5:** The correlation analysis between lipids, lipoproteins, and apoA-I contents of HDL subclasses (Controlling for TG)

	TC		LDL-C		HDL-C		ApoA-I		ApoB-100	
	**t**	***P***	**t**	***P***	**t**	***P***	**t**	***P***	**t**	***P***

**Preβ**_**1**_**-HDL**	.390	.000	.381	.000	.063	.169	.491	.000	.183	.000
**Preβ**_**2**_**-HDL**	.136	.003	.077	.089	.165	.000	.489	.000	-.008	.867
**HDL**_**3c**_	.217	.000	.156	.001	.193	.000	.360	.000	.112	.013
**HDL**_**3b**_	.296	.000	.256	.000	.140	.002	.523	.000	.104	.022
**HDL**_**3a**_	.091	.045	.075	.100	.058	.204	.567	.000	.045	.322
**HDL**_**2a**_	-.130	.004	-.128	.005	.200	.000	.558	.000	-.031	.494
**HDL**_**2b**_	-.136	.003	-.216	.000	.286	.000	.415	.000	-.168	.000

TG, triglyceride; TC, total cholesterol; LDL-C, low density lipoprotein cholesterol, ApoA-I, B-100, apolipoproteinA-I, B-100; HDL_3c_, _3b_, _3a_, _2a_, _2b_, high density lipoproteins_3c_, _3b_, _3a_, _2a_, _2b_.

**Table 6:** *P *values calculated by 2-way ANOVA for HDL subclasses

	Factor A (TC)	Factor B (TG)	Factor interaction (TC × TG)
**Preβ**_**1**_**-HDL**	.000	.000	.000
**Preβ**_**2**_**-HDL**	.002	.042	.003
**HDL**_**3c**_	.000	.192	.084
**HDL**_**3b**_	.000	.369	.296
**HDL**_**3a**_	.279	.001	.006
**HDL**_**2a**_	.665	.000	.217
**HDL**_**2b**_	.000	.000	.000

ANOVA, analysis of variance; TC, total cholesterol; TG, triglyceride; HDL_3c_, _3b_, _3a_, _2a_, _2b_, high density lipoproteins_3c_, _3b_, _3a_, _2a_, _2b_.

## Discussion

Numerous observational studies have demonstrated that a strong, continuous, graded, and independent association between cholesterol and the risk of CHD. A high TC is a marker for atherogenic lipoproteins. The elevated plasma TC contributes to coronary atherosclerosis throughout life; which measured in young adulthood correlate with CHD rates later in life and over a lifetime. Increased lifetime risks associated with high TC levels ( ≥ 6.21 mmol/L) are clearly evident and justify clinical therapies to reduce long-term risk. But even borderline-high TC (5.17-6.18 mmol/L) carries significant long-term risk, and it deserves clinical intervention [18-22]. In this regard, only TC levels < 5.17 mmol/L was expectation for Western people.

Population studies have shown a highly consistent, inverse correlation between plasma concentrations of HDL-C and atherosclerotic cardiovascular disease risk in humans [23] and the concept that certain subfractions of HDL may be better predictors of cardiovascular risk is attractive [11]. Asztalos *et al. *[24] found that large α-1 HDL (HDL_2b_) was most significantly associated with CHD prevalence, and their study demonstrated that each milligram per decaliter increase in α-1 HDL level decreased the odds of CHD by 26% in a model including all established CHD risk factors. Thus, HDL lipoprotein subfraction tests may be useful to help determine the need to treat patients at intermediate risk for CHD as determined by more conventional tests [25-32]. Epidemiological investigations have established that a high level of LDL-C is a major risk factor for developing CHD. For persons without other CHD risk factors, risk for CHD is relatively low when LDL-C levels are <3.34 mmol/L. However, although the availability the effective of LDL-C lowering therapies, many patients continue to develop CHD and the contributions of other plasma lipids and lipoprotein subclasses to the risk of CHD is increasingly being recognized.

In current work, for grouped analyses, individuals were classified according to approximately equal ninths of baseline TC for the entire study population (Figure 1). Trends in mean values of major HDL subclasses (preβ_1_-HDL and HDL_2b_) across these ninths were assessed through simple linear regression, in these models with the contents of preβ_1_-HDL and HDL_2b _as the dependent variable and the levels of TC as independent variable. The Figure 1 showed that the levels of TC were liner with HDL_2b _contents and HDL_2b _contents can be reduced 17 mg/L for 0.5 mmol/L increment in TC. At the same time, according to ATP-III guidelines, the Chinese subjects were divided into desirable TC (<5.17 mmol/L), borderline-high TC (5.17-6.18 mmol/L), and high TC ( ≥ 6.21 mmol/L) groups, and observed HDL subclasses distribution profile on different TC levels that is the particles size of HDL subclasses shifted towards smaller with increased TC levels. These findings suggested that elevated TC blocked the maturation of HDL subclasses metabolism and the efficiency of reverse cholesterol transport (RCT) might be impeded.

Numerous investigations have documented that in hyperolesterolemic subjects, Lecithin:cholesterol acyltransferase (LCAT) activity was low while cholesterol ester transport protein (CETP) activity was high, which was associated with the increased plasma TC level in these persons [33]. The activity of LCAT is required for normal plasma lipoprotein structure and is instrumental in HDL remodeling. LCAT may catalyze unesterified cholesterol to cholesterol ester (CE) and promote the conversion of preβ_1_-HDL and HDL_3 _to HDL_2_. CETP is a plasma hydrophobic glycoprotein made by liver and adipose that circulates in the plasma bound to lipoproteins. It mediates exchange of core lipids between very low density lipoprotein (VLDL)-TG, LDL-TG and HDL-CE. The net effect of CETP action on HDL is depletion of CE and enrichment with TG, with an overall net reduction in the size of HDL particles [34].

Furthermore, we surprisingly found the distribution of HDL subclasses have shown abnormality characterized by the contents of HDL_2b _(324.2 vs 379.9 mg/L) decreased and the contents of preβ_1_-HDL (90.4 vs 76.5 mg/L) increased significantly for desirable TC Chinese subjects compared with those for normolipidemic Chinese subjects in our previous study [9]. Why the distribution of HDL subclasses has reversed for subjects in desirable TC levels when the mean concentrations of TG, TC, and LDL-C kept the relative normal range.

Our results exhibited that there were 103 subjects with TG < 2.26 mmol/L and 107 subjects with HDL-C ≥ 1.03 mmol/L in the 176 desirable TC subjects as well as the characteristic of distribution in HDL subclass distribution for these two groups subjects was consistent with that for normolipidemic subjects [9]. On the contrary, profile of HDL subclass distribution for subjects with TG ≥ 2.26 mmol/L showed a reduced in contents of HDL_2b _(from 381.9 to 242.8 mg/L), but an increased in contents of preβ_1_-HDL (from 79.3 to118.1 mg/L) compared to the subjects with TG < 2.26 mmol/L in desirable TC levels. Similarly, the distribution of HDL subclasses for subjects with HDL-C < 1.03 mmol/L might be reversed characterized by small-sized preβ_1_-HDL elevated while large-sized HDL_2 _declined.

The decreased lipoprotein lipase (LPL) and phospholipids transfer protein (PLTP) activities along with elevated hepatic lipase (HL) activity are frequently observed in higher TG levels and lower HDL-C levels. LPL cleanses triglyceride-rich lipoproteins (TRL) from circulation through its role in hydrolysis of TGs in chylomicron (CM) and VLDL. PLTP favors the formation of larger-sized HDL particles. Depletes the core of large HDL_2 _by HL and helps to form smaller HDL_3 _as well as lipid-free apoA-I and/or preβ-HDL. In consequence of the changes in activities of lipoprotein-modifying plasma enzymes, which resulted in the particle size of HDL subclasses tend to small. Low levels of HDL-C and elevated TG levels were associated with a high incidence of CHD. For clinical purposes, low HDL-C (<1.03 mmol/L) plus elevated TG (≥ 2.26 mmol/L) define atherogenic dyslipidemia. The relationship between HDL-C and CHD remained after adjustment for age and TG levels; where even small differences in the level of HDL-C are associated with substantial variations in the risk of major coronary events. It has been documented that cholesterol efflux is well correlated with plasma HDL-C concentrations. Data from the Framingham population indicated that, at any given level of TC, the relative risk of CHD increases with decreasing levels of HDL-C [35]. Asztalos *et al.*[24] considered that altered HDL subclasses in low HDL-C subjects were prone to CHD by RCT.

It is well known that the most common hyperlipidemia for the Chinese population was characterized by elevated TG levels (ie, hypertriglyceridemia [HTG]). Our previous study displayed that HTG accounted for about 61% of total hyperlipidemia [11]. Liu suggested that HTG in China was induced by high-carbohydrate diets of the populations. High-carbohydrate diets may result in increased concentration of plasma glucose and, thus, high-insulin levels. Hyperinsulinemia stimulates the production and secretion of TG and VLDL, which lead to HTG [36].

Because of the elevated TG concentrations usually not only accompanied with reduced HDL-C, it also carried increased LDL-C concentrations. Make use a cut-point value of 3.34 mmol/L for the LDL-C to further dichotomize the Chinese subjects in desirable TC levels. The data presented that about 20 subjects with LDL-C ≥ 3.34 mmol/L in desirable TC levels, and the particle size of HDL subclass for these subjects tend to small. Increased levels of plasma LDL-C resulted in decreased CETP activity might be explained the alteration of HDL subclasses in subjects with LDL-C ≥ 3.34 mmol/L [37]. We also observed that among the 156 subjects with LDL-C < 3.34 mmol/L, one third (52) subjects with TG ≥ 2.26 mmol/L-HDL-C < 1.03 mmol, and the contents of HDL_2b _(215.0 mg/L) reduced significantly along with those of preβ_1_-HDL (122.7 mg/L) elevated significantly for these subjects. Collectively these findings demonstrated that distribution of HDL subclasses has reversed in desirable TC levels, especially in Chinese population.

## Conclusions

Briefly, the higher TC levels, the particles size of HDL subclasses tend to small suggested that elevated TC blocked the maturation of HDL subclasses metabolism and impeded the efficiency of RCT. The TC was liner with HDL_2b _contents and HDL_2b _contents can be reduced 17 mg/L for 0.5 mmol/L increment in TC levels. The phenotype of HDL subclasses distribution was not expectation for Chinese Population with desirable TC in accordance with the ATP-III guidelines for TC levels. Thus, from the HDL subclasses distribution point, when assessing the CHD risk not only rely on the TC levels, but also the concentrations of TG, HDL-C and LDL-C must considered in case the potential risk for desirable TC subjects with other plasma lipids metabolism disorders.

## The abbreviations used are

HDL: high density lipoproteins; TC: total cholesterol; ATP-III: Adult Treatment Panel-III; TG: triglyceride; LDL-C: low density lipoprotein cholesterol; CHD: coronary heart disease; HRP: horseradish peroxidase; IgG: immunoglobulin G; ANOVA: analysis of variance; RCT: reverse cholesterol transport; LCAT: lecithin:cholesterol acyltransferase; CETP: cholesterol ester transport protein; CE: cholesterol ester; VLDL: very low density lipoprotein; LPL: lipoprotein lipase; PLTP: phospholipids transfer protein; HL: hepatic lipase; TRL: triglyceride-rich lipoproteins; CM: chylomicron; HTG: hypertriglyceridemia.

## Conflict of interests statement

The authors declare that they have no competing interests.

## Authors' contributions

LT participated in the design of study and manuscript preparation along with editing. YHL conceived of the study, and helped to review the manuscript. MDF participated in manuscript reviewing and drafting. SYL performed the data acquisition and analysis. YHX performed data and statistics analysis. LQJ participated in drafted the manuscript. All authors read and approved the final manuscript.

## Notice of grant support

This work supported by the grants from National Natural Science Foundation of China (Grant No. 30800474) and the Fundamental Research Funds for the Central Universities (Grant No. 2010SCU11029).
